# Trends and Spatiotemporal Patterns of Avian Influenza Outbreaks in Italy: A Data-Driven Approach

**DOI:** 10.3390/idr16010001

**Published:** 2023-12-19

**Authors:** Francesco Branda, Sandra Mazzoli, Massimo Pierini, Massimo Ciccozzi

**Affiliations:** 1 Unit of Medical Statistics and Molecular Epidemiology, Università Campus Bio-Medico di Roma, 00128 Rome, Italy; 2 Website of the Epidemiological Group, EpiData.it, 24121 Bergamo, Italy; 3 Statistics and Big Data, Universitas Mercatorum, 00186 Rome, Italy

**Keywords:** avian influenza, bird flu, HPAI, H5N1, poultry, wild birds, public health, virus surveillance, open data, epidemiology

## Abstract

In recent years, the unprecedented spread of the Avian Influenza Viruses (AIVs) among birds and mammals has caused devastation in animal populations, including poultry, wild birds, and some mammals, damaging farmers’ livelihoods and the food trade. Given the urgency of the situation, it is particularly important that scientists and the public can access research results and data as soon as possible. The main aim of this study is to present a global open-access dataset of Avian Influenza outbreaks to enable researchers and policymakers (i) to rapidly detect, and respond to animal outbreaks as the first line of defense; (ii) to conduct epidemiological and virological investigations around animal outbreaks and human infections; and (iii) to communicate the risk. We show the potential use of this dataset to the research community by analyzing the most updated information on past and current Highly Pathogenic Avian Influenza (HPAI) outbreaks in domestic poultry and wild birds over the period from October 2021 to July 2023 in Italy. In addition, we applied indices borrowed from Economics (such as Homogeneity, Specialization, and Location Index) to the wild birds dataset to show their possible usage in epidemiology.

## 1. Introduction

The devastating consequences of COVID-19 on human health have stimulated extensive discussions on improving preparedness and defenses against future pandemics. In recent times, fears have risen about the potential emergence of a new pandemic caused by Avian Influenza (AI), i.e., a highly contagious viral disease primarily affecting birds that is caused by a virus of the *Orthomyxoviridae* family. AI may be classified as Low Pathogenic Avian Influenza (LPAI) or High Pathogenic Avian Influenza (HPAI) [1], according to the molecular characteristics of the Avian Influenza Virus (AIV) involved and its ability to cause disease and mortality in chickens.

The current global epidemic of HPAI among animals is due to a distant descendant of the original influenza A(H5N1) virus that emerged in 1996 and caused human outbreaks of Avian Influenza in the past [2,3]. Moving along bird migration routes to Europe and the Americas, this new Highly Pathogenic strain of Avian Influenza A(H5N1) virus has led to an unprecedented number of deaths in wild birds and poultry. Recently, more and more outbreaks have been reported among mammals [4,5,6], which are biologically closer to humans than birds, raising concerns that the virus may adapt to infect humans more easily. However, based on the available information [7], human cases remain very rare [8] and those reported are mostly related to close contact with infected birds and contaminated environments [9,10].

In such a complex epidemiological context, it is particularly important that the research results and data are stored in appropriate public repositories to support the ongoing public health emergency response efforts.

The methodology used in this study consists of several steps. First, unstructured data are extracted from government sources or public health institutions (e.g., peer-reviewed scientific papers [11], bulletins [12]) using an automated script that continuously and systematically collects the required data. Then, high-density areas (called hotspots) are identified to assess the spatial dynamics of outbreaks to inform real-time response and enable evidence-based decision-making. Finally, economics-derived indices are used to investigate and understand outbreak patterns to strategize interventions.

To show the potential use of our dataset to the research community, we present here the analysis of HPAI outbreaks in domestic poultry and wild birds over the period from October 2021 to July 2023 in Italy. The results of the experimental evaluation show the effectiveness of the proposed approach, providing a direct visual assessment of the geographic distribution of risk areas at region- and province-level in Italy. We find evidence of the existence of spatial clusters of high incidences in Lombardy, Veneto, and Emilia-Romagna. Moreover, our experimental investigation of the use of economics-derived indices in epidemiology produced some interesting results. For example, Black-headed gull is the most homogeneous species (Homogeneity Index (HI): 44); Emilia-Romagna and Veneto are the less homogenous regions (HI: 3.41, 10.20); less specialized regions are Veneto, Lombardia and Emilia-Romagna (Specialization Index (SI): 15.72, 24.53, 28.62).

The rest of the paper is organized as follows. Section 2 describes the proposed methodology. Section 3 presents the experimental evaluation of the methodology on a case study. Finally, Section 4 concludes the paper.

## 2. Materials and Methods

**Figure 1:**
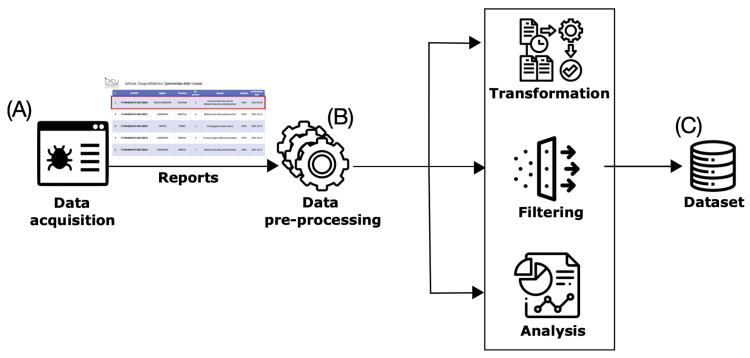
Main steps of the proposed methodology.

This section describes the methodology that we have designed to discover spatiotemporal patterns from Avian Influenza data. Figure 1 provides a schematic overview of the proposed methodology.

The input data of the analysis is the set of collected bulletins from the Istituto Zooprofilattico Sperimentale delle Venezie (IZSV) web page [12] to be processed (stage A). Once the bulletins were collected, stage (B) was performed to make data suitable for analysis. First, we extracted the following information and entered into a structured template within a dedicated .csv file: (i) geographic information, i.e., details about the location of the Avian Influenza outbreaks in both domestic poultry and wild birds; (ii) temporal information, i.e., key dates in the management of Avian Influenza events, such as the confirmation date, which signifies when an outbreak was officially confirmed, the extinction date, indicating when control measures successfully contained the outbreak, and the dates when protection and surveillance zone measures were enforced and later revoked; (iii) affected animals species, i.e., data related to the number and types of animals affected, as well as the specific HPAI subtype associated with reported cases.

Then, we transformed data by enriching geographic information according to the National Institute of Statistics (ISTAT) nomenclature to enable us to use region and province codes as key variables to exploit the data more easily in statistical software such as R [13]. Moreover, species needed to be improperly aggregated because (i) they have been aggregated in the original dataset and could not be ungrouped; (ii) many of them have null or very low counts. This aggregation is not optimal because it does not take into account genus, family, and order. Furthermore, concentration indices should be applied to non-pooled datasets in order to ensure the maximum informative level. Nevertheless, the usage of economics-derived indices with this dataset is only illustrative of their applicability in epidemiology and potentially valuable insights that can be disclosed. Species have been grouped into three categories:

A. Black-headed gull (*Chroicocephalus ridibundus*).
B. Common teal (*Anas crecca*), Mallard duck (*Anas platyrhynchos*), Laying hen (*Gallus gallus domesticus*), Rock dove (*Columba livia*), Common peacock (*Pavo cristatus*).
C. Greylag goose (*Anser anser*), Eurasian wigeon (*Mareca penelope*), Mute swan (*Cygnus olor*), White stork (*Ciconia ciconia*), Northern pintail (*Anas acuta*), Great Spotted Woodpecker (*Dendrocopos major*), Common buzzard (*Buteo buteo*), Greater flamingo (*Phoenicopterus roseus*), Mandarin duck (*Aix galericulata*), Greater white-fronted goose (*Anser albifrons*), Eurasian collared dove (*Streptopelia decaocto*), Grey heron (*Ardea cinerea*), Peregrin falcon (*Falco peregrinus*), Common kestrel (*Falco tinnunculus*), Yellow-legged gull (*Larus michahellis*), Tawny owl (*Strix aluco*), Anatidae (unidentified), Little egret (*Egretta garzetta*), Common woodpigeon (*Columba palumbus*), Eurasian oystercatcher (*Haematopus ostralegus*), Laridae, Carrion Crow (*Corvus corone*).

Finally, we performed spatial analysis and incorporated economics-derived indices into the analysis to provide valuable insights into the geographic distribution of Avian Influenza dynamics. Specifically, we used the Getis-Ord Gi* statistics [14] to identify specific geographic areas with statistically significant patterns and by examining metrics such as Specialization, we can identify regions where Avian Influenza cases are less heterogeneous with respect to the marginal global territory counts. This allows us to pinpoint areas where disease control measures should be intensified. Homogeneity, another essential index, sheds light on the diversity of Avian Influenza cases within each single region. Understanding homogeneity helps us gauge the potential ripple effects of an outbreak. Location, a key spatial index (LI), aids in deciphering the geographical pathways of disease spread. Regions with strong ties and proximity might facilitate rapid transmission, necessitating targeted surveillance and intervention efforts. By analysing these indices, we can strategically allocate resources and prioritize areas most susceptible to impact.

**Figure 2:**
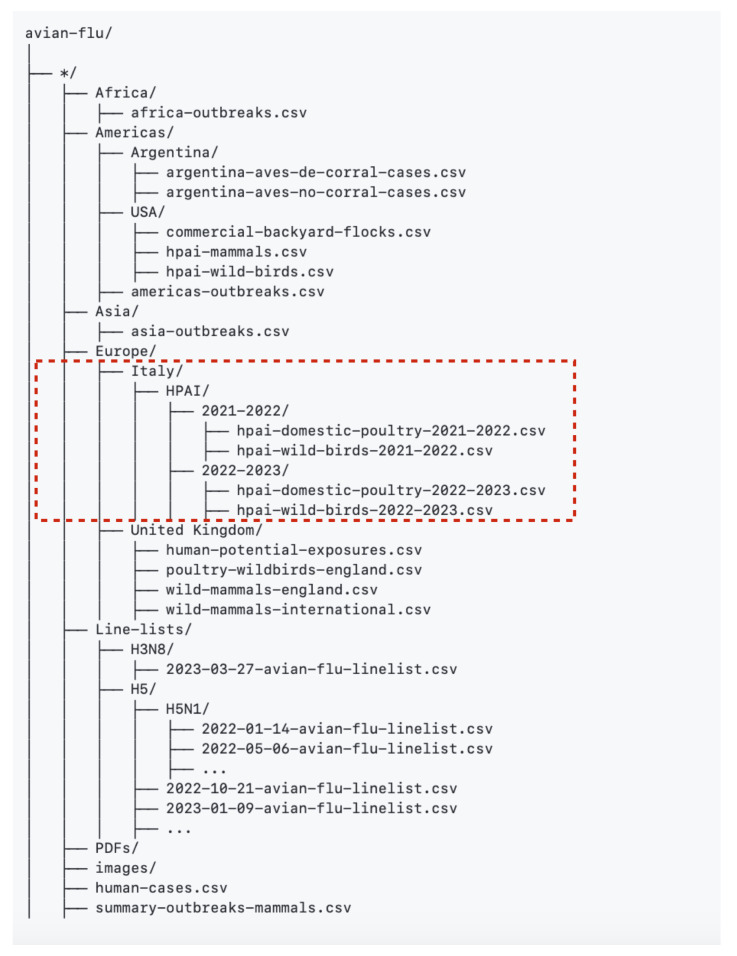
Schematic structure of the Open Avian Flu Dataset (OAFD) dataset.

The schematic structure of the resulting dataset (stage C) is shown in Figure 2. The red dashed line highlights only the data fields used for the purpose of this paper, i.e., “hpai-domestic-poultry-yyyy.csv” (Table 1), and “hpai-wild-birds-yyyy.csv” (Table 2), which contain information related to HPAI in domestic poultry and wild birds, respectively, for a specific time period, as indicated by the “yyyy” in the file name. A live version of the dataset is publicly available on the GitHub repository at the link https://github.com/fbranda/avian-flu/tree/main/Europe/Italy/HPAI (accessed on 21 July 2023). A static version of the dataset can also be found on Zenodo [15], which includes a version of the dataset at the time of submission, running from October 2021 to 20 July 2023.

### Usage Notes

As an example of data use, we show how this dataset can be a valuable resource for epidemiologists and public health authorities for tracking and understanding Avian Influenza outbreaks across different regions using the Getis-Ord Gi* statistics. It operates on the principle of spatial autocorrelation, which examines the extent to which a variable’s values are clustered or dispersed in geographic space. Unlike global spatial autocorrelation measures (e.g., Moran’s I [16]), which provide a single value to describe the overall spatial pattern, Getis-Ord Gi* focuses on local patterns. It calculates statistics for each feature (e.g., points or areas) in the dataset, helping to identify specific locations with statistically significant clusters of high or low values. A brief description of how the Getis-Ord Gi* statistics works is the following:

1. Select a study area and define the spatial scale: Choose the geographic area of interest and determine the spatial scale, such as the radius of the neighborhood surrounding each feature. The spatial scale defines the size of the window used to assess local clustering.
2. Calculate the local sum: For each feature in the dataset, calculate a local sum by summing the values of the feature and its neighboring features within the defined spatial scale.
3. Calculate the local mean and standard deviation: Compute the local mean and standard deviation of the feature values within the neighborhood. The local mean represents the average value in the neighborhood, while the standard deviation measures the variation within that neighborhood.
4. Compute the Getis-Ord Gi* statistics: The Getis-Ord Gi* statistics is calculated for each feature using the formula:
  $$Gi*=\frac{\sum_{j=1}^{n}w_{i,j}x_{j}-\bar{x}\sum_{j=1}^{n}w_{i,j}x_{j}}{S\sqrt{\frac{n\sum_{j=1}^{n}w_{i,j}^{2}-{\left(\sum_{j=1}^{n}w_{i,j}\right)}^{2}}{n-1}}}$$
  where Gi* represents the Getis-Ord Gi* statistics for a specific feature (point or area) in the dataset, $w_{i,j}$ represents a spatial weight matrix that defines the relationships between the feature being analyzed (*i*) and its neighboring features (*j*) within a defined spatial scale, $x_{j}$ is the value of the feature being analyzed, $\bar{x}$ is the mean value of the variable of interest across all features, *n* is the total number of features in the dataset, and *S* is the standard deviation of the variable of interest across all features.
5. Assess significance: Calculate the Z-score [17] for each feature’s Getis-Ord Gi* statistics. The Z-score measures how many standard deviations the local sum is from the mean. High positive Z-scores indicate hotspots (areas with significantly high values), while low negative Z-scores indicate coldspots (areas with significantly low values).
6. Generate a local cluster map: Visualize the results on a map. Features with high positive Z-scores are depicted as hotspots, while those with low negative Z-scores are shown as coldspots.

Since the time series are characterized by too many null frequencies in our dataset and the infection dates are unknown, the data can be considered nominal summing up the counts of one or more years. We summed up by species and regions the counts of 2022 and 2023 and, for a descriptive analysis, applied indices such as Homogeneity Index, Specialization Index, and Location Index [18].

The usage of these indices in fields other than economics is, in our opinion, only a matter of correct interpretation and limitations analysis (i.e., understanding exactly what the indices cannot reveal given the data set to which they have been applied) rather than applicability. Thus, we will briefly discuss each index we used in this descriptive analysis. Hereafter, we will refer to a matrix of nominal data counts with *R* rows and *C* columns, where each of the two variables (rows and columns) are labeled into mutually exclusive categories, namely “species” and “regions” and where $n_{rc}$ is the element of the *r*-th row and the *c*-th column.

Homogeneity Index is an absolute index that, for each row or column and marginal counts, measures the degree of the distribution homogeneity, from the maximum (*HI* = 100, all counts are concentrated in a single category) to the minimum (*HI* = 0, all categories are equally represented) [18]. Here, we have used the more common *HI* with α = 2 but we recommend further research about the usage of *HI* with a higher α parameter in epidemiology. The Homogeneity Index of the *r*-th row can be defined as

(1) $$HI_{r}=\frac{C\sum_{c=1}^{C}{\left(\frac{n_{rc}}{n_{r.}}\right)}^{2}-1}{C-1}\cdot 100$$

where $n_{rc}$ represents the value or quantity of Avian Influenza cases in the region *r* for category *c*, $n_{r.}$ is the total number of Avian Influenza cases in the region *r* across all categories, and *C* is the total number of categories.

Specialization Index is a relative index that, for each row or column, measures the degree of the distribution deviation from the marginal distribution which is taken as a benchmark. It ranges from *SI* = 0 (no specialization, i.e., the category is distributed exactly as the marginal count) to *SI* = 100 (maximum specialization, i.e., maximum dissimilarity degree between the category’s and the marginal distribution) [18].

(2) $$SI_{r}=\frac{1}{2}\sum_{c=1}^{C}\left|\frac{n_{rc}}{n_{r.}}-\frac{n_{.c}}{n_{..}}\right|\cdot 100$$

where $n_{rc}$ represents the value or quantity of Avian Influenza cases in region *r* for category *c*, $n_{r.}$ is the total number of Avian Influenza cases in region *r* across all categories, $n_{.c}$ is the total number of Avian Influenza cases in category *c* across all regions, $n_{..}$ is the total number of Avian Influenza cases across all regions and categories, and *C* is the total number of categories.

Location Index is a relative index that, for each matrix element count, measures the odds of local concentration with respect to the marginals, taken as a benchmark. If 0 ≤ *LI* < 1 the element is less concentrated than the benchmark; if *LI* > 1 the element is more concentrated than the benchmark; if *LI* = 1 the element is equally concentrated as the benchmark [18].

(3) $$LI_{rc}=\frac{n_{rc}/n_{r.}}{n_{.c}/n_{..}}$$

where $n_{rc}$ represents the value or quantity of Avian Influenza cases in region *r* for category *c*, $n_{r.}$ is the total number of Avian Influenza cases in region *r* across all categories, $n_{.c}$ is the total number of Avian Influenza cases in category *c* across all regions, and $n_{..}$ is the total number of Avian Influenza cases across all regions and categories.

## 3. Results

To assess the reliability of the generated dataset, we conducted an experimental investigation of the risk mapping of Avian Influenza events in Italy, focusing on domestic poultry and wild birds. The goal of our analysis comprises identifying geographic areas with a high concentration of disease outbreaks and determining the specific species that are affected within that region, which can help to better understand the spatial variation of the disease and communicate the risk.

**Figure 3:**
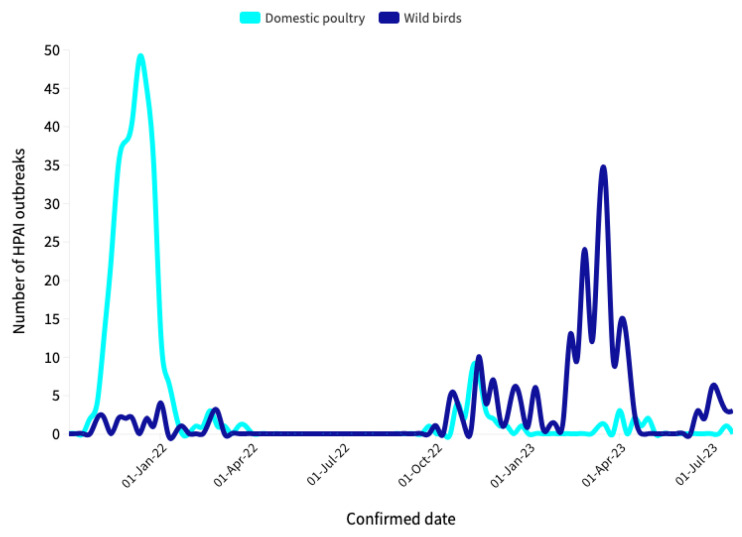
Avian Influenza outbreaks in domestic poultry and wild birds, by confirmed data, Italy, 19 October 2021–20 July 2023.

Figure 3 provides a preliminary view of the collected data, offering valuable insights into data trends. The plot immediately reveals several interesting features. First and foremost, there is a noticeable decline in the number of Avian Influenza events in domestic poultry over the specified time period, indicating a clear overall decreasing trend in the data. Secondly, the plot highlights the HPAI epidemic observed in wild birds during the 2022–2023 epidemiological year. Although this epidemic is still ongoing, it has already surpassed the previous epidemiological year (2021–2022) in terms of the total number of HPAI virus detections reported in wild birds. Specifically, there have been 239 reported detections in the current year, in contrast to the 23 reported in the previous year. This significant increase underscores the severity of the ongoing outbreak in wild bird populations and the need for continuous monitoring and intervention measures to limit the exposure of farmed birds to wild birds in order to reduce the risk of introducing Avian Influenza to farms.

As summarized in Table 3, a total of 620 outbreaks of HPAI A(H5N1) virus were reported from 19 October 2021, to 20 July 2023. 358 outbreaks were detected in domestic poultry, of which 282 in the northeastern regions (n = 273 in Veneto; n = 7 in Emilia-Romagna; n = 2 in Friuli-Venezia Giulia), 70 in the northwest regions (Lombardy n = 69; Piedmont n = 1), and 6 in the central regions (Tuscany n = 5; Lazio n = 1). As for wild birds, 262 outbreaks were detected. Again, northeastern regions were the most affected (n = 163, of which n = 76 in Veneto, n = 61 in Emilia-Romagna, n = 10 in Friuli-Venezia Giulia and n = 16 in Autonomous Province (AP) of Trento), followed by the northwest regions (n = 89, of which n = 85 in Lombardy and n = 4 in Piedmont), the central regions (n = 3, of which n = 2 in Umbria and n = 1 in Lazio), and the southern regions (n = 7, of which n = 4 in Campania, n = 2 in Sardinia and n = 1 in Apulia).

**Figure 4:**
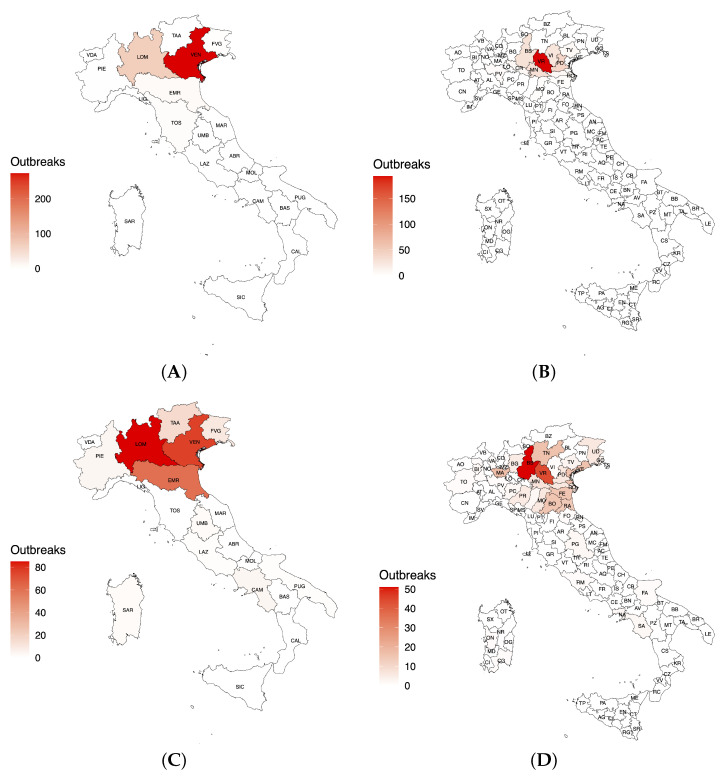
Map of the main outbreaks detected during the HPAI 2021–2023 epidemic in Italy by (**A**) domestic poultry per region; (**B**) domestic poultry per province; (**C**) wild birds per region; (**D**) wild birds per province.

The spatial distribution of Avian Influenza outbreaks discovered through our analysis is shown in Figure 4, which includes two panels: in the left panel, a regional-level overview is presented, showing trends in outbreaks in different geographic areas. On the other hand, the right panel highlights the provinces that have been hardest hit by these outbreaks in the regions identified in the first panel. Considering the domestic poultry species, Figure 4A shows two notable clusters of regions, distinctly identifiable through varying colors: Veneto (in red) and Lombardy (in pink), i.e., signifying regions with high and moderate outbreak densities, respectively. In the reporting timeframe, most domestic poultry outbreaks were documented in Verona (n = 193), Padua (n = 41), Mantua (n = 29), Brescia (n = 28), and Vicenza (n = 25) (Figure 4B). HPAI outbreaks in wild birds were primarily observed in Lombardy, Veneto, and Emilia-Romagna (Figure 4C), of which Brescia (n = 49), Verona (n = 39), Ferrara and Bologna (n = 15) were the most affected provinces, respectively (Figure 4D).

**Figure 5:**
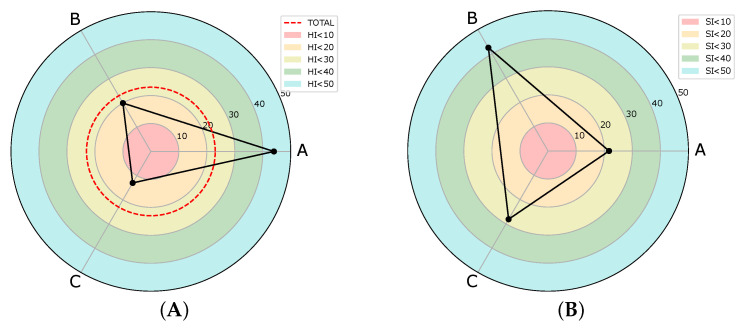
(**A**) Homogeneity and (**B**) Specialization Index by species.

Several interesting insights into the use of economics-derived indices in epidemiology can be drawn from our experimental investigation:

1. Even if species A are the highest in Veneto, they are less localized than the benchmark, i.e., with respect to the marginal counts they are less concentrated (Table 4).
2. Excluding obvious results (Friuli-Venezia Giulia, Sardinia and Umbria) species B and C are more localized in Emilia-Romagna than Lombardy and Veneto (Table 5).
3. Even if species A are the most homogeneous (Figure 5) because their distribution is mostly concentrated in Lombardia, the most specialized (i.e., mostly different from the marginal counts benchmark) are species B.
4. The less homogeneous (i.e., the most uniformly distributed) regions are Emilia-Romagna and Veneto (Table 6).
5. The last specialized regions are Veneto, Emilia-Romagna and Lombardy (Table 7).

These evaluations don’t take into account the distribution of the species over the territory, which should be considered and compared with the Avian Influenza cases. These are only simple examples of useful insights that can be achieved by applying concentration indices to a nominal epidemiological dataset. The low counts, the presence of many null frequencies, and the need to aggregate many different species do not allow a deeper analysis in this particular case.

## 4. Discussion

This paper presented an extensive overview of HPAI virus detections in domestic poultry and wild birds in Italy between October 2021 and July 2023. Based on the bulletins published by IZSVe, the experimental evaluation provided comprehensive information regarding the impacted regions and the most affected species. The choice of the IZSV as the primary source of data for our analysis stems from several strategic considerations. First, the IZSV consistently provides well-organized and regularly updated data, which are essential for maintaining the relevance and timeliness of our analysis. Second, the IZSV is renowned for its commitment to the scientific integrity and reliability of its data. Using data from such a highly reputable institution ensures the credibility and robustness of our analysis.

The study revealed two completely different scenarios. During the 2021–2022 epidemiological year, there was a significant number of HPAI virus detections in domestic poultry, mainly affecting Veneto and Lombardy. On the other hand, in the 2022–2023 epidemiological year, which began on 22 September 2022 with the first case detected in poultry by the National Reference Laboratory (NRL) for Avian Influenza and Newcastle Disease, it is important to note that as the occurrences in poultry decreased significantly, the number of cases in wild birds has shown a marked increase, especially in Lombardy, Veneto, and Emilia-Romagna, underscoring the adaptability and variable impact of Avian Influenza on different animal populations.

The indices we applied have been originally conceived in the field of Economics. Nevertheless, their technical definitions allow applications into other fields whenever absolute frequencies of categorical variables are available. They can help identifying datasets’ characteristics not directly visible. In this study, we present a first innovative application in zoological epidemiology. We wish to remark that, despite their origin, these indices applied to the described dataset should not be interpreted with a socioeconomic meaning but in terms of geographical and cross-species distribution of Avian Influenza in Italian regions. We strongly recommend further studies to deeply investigate other possible fields of application, especially in epidemiological contexts. Three major limitations can be detected for the usage of these indices in the dataset: (i) not all Italian regions are represented since many territories have got zero counts; thus, the benchmark does not correspond with the entire national territory but with the selected regions only; (ii) several species needed to be aggregated because (a) they have been aggregated in the analyzed reports and could not be ungrouped; (b) the counts of single species were too low and would have raised extreme values of relative indices; (iii) because of low counts and unknown infection date (the date in the reports refers to the confirmation), the counts of both 2022 and 2023 have been summed up by species and regions.

In future work, other research issues may be investigated. First, we may further expand the scope and depth of data collection. This expansion may involve incorporating data from additional sources to create more comprehensive datasets for analysis. Second, we will explore the application of disease risk models for forecasting regional trends. In particular, we are interested in studying the application of Bayesian hierarchical modeling in spatial epidemiology, which involves the integration of data from various levels of spatial aggregation, such as health data, regional-level data, and demographic information. This approach will allow us to make more accurate predictions about the trend of Avian Influenza outbreaks and identify potential risk factors. Third, in an increasingly interconnected world, the global spread of Avian Influenza accentuates the need for international collaboration. Future research can focus on developing international frameworks and protocols to enable rapid integration among multiple research groups and governments. A truly open platform can help users overcome geographic, organizational, and social barriers to accessing information.

## Figures and Tables

**Table 1:** Structure of the file “hpai-domestic-poultry-yyyy.csv” within the folder Italy.

Variable	Description	Format
n	Incremental sequence numbers assigned at the time of the creation/update of the dataset	String
ADIS Reference	Unique identifier alpha-numeric code for reported outbreak	Numeric
Region	Region name	String
Code region	Region 2-digit code	Numeric
Province	Province name	String
Code province	Province 3-digit code	Numeric
Abbreviation province	Province 2-letter code	String
Latitude	Latitude of the province	Numeric
Longitude	Longitude of the province	Numeric
Species	Species name of reported cases	String
HPAI strain	HPAI subtype name	String
Confirmation date	Date of confirmation of the event	yyyy-mm-dd
Extinction date	End date of event control	yyyy-mm-dd
Measures Protection Zone in force until	Boundaries of protection zone and the date of revocation of the measures applied	yyyy-mm-dd
Measures Surveillance Zone in force until	Boundaries of surveillance zone and the date of revocation of the measures applied	yyyy-mm-dd

**Table 2:** Structure of the file “hpai-wild-birds-yyyy.csv” within the folder Italy.

Variable	Description	Format
n	Incremental sequence numbers assigned at the time of the creation/update of the dataset	String
ADIS Reference	Unique identifier alpha-numeric code for reported outbreak	Numeric
Region	Region name	String
Code region	Region 2-digit code	Numeric
Province	Province name	String
Code province	Province 3-digit code	Numeric
Abbreviation province	Province 2-letter code	String
Latitude	Latitude of the province	Numeric
Longitude	Longitude of the province	Numeric
No. animals	Number of infected animals in the outbreak	Numeric
Species	Species name of reported cases	String
HPAI strain	HPAI subtype name	String
Confirmation date	Date of confirmation of the event	yyyy-mm-dd

**Table 3:** Total number of the HPAI A(H5N1) animal outbreaks reported by IZSVe, from October 2021 to July 2023.

Macro-Area	Region	Year	Domestic Poultry	Wild Birds
South/Islands	Abruzzo	2021–2022	0	0
		2022–2023	0	0
	Apulia	2021–2022	0	1
		2022–2023	0	0
	Basilicata	2021–2022	0	0
		2022–2023	0	0
	Calabria	2021–2022	0	0
		2022–2023	0	0
	Campania	2021–2022	0	4
		2022–2023	0	0
	Molise	2021–2022	0	0
		2022–2023	0	0
	Sardinia	2021–2022	0	0
		2022–2023	0	2
	Sicily	2021–2022	0	0
		2022–2023	0	0
Center	Lazio	2021–2022	1	1
		2022–2023	0	0
	Marche	2021–2022	0	0
		2022–2023	0	0
	Tuscany	2021–2022	4	0
		2022–2023	1	0
	Umbria	2021–2022	0	0
		2022–2023	0	2
North-East	AP Bolzano	2021–2022	0	0
		2022–2023	0	0
	AP Trento	2021–2022	0	0
		2022–2023	0	16
	Emilia-Romagna	2021–2022	2	1
		2022–2023	5	60
	Friuli-Venezia Giulia	2021–2022	1	1
		2022–2023	1	9
	Veneto	2021–2022	248	9
		2022–2023	25	67
North-West	Aosta Valley	2021–2022	0	0
		2022–2023	0	0
	Liguria	2021–2022	0	0
		2022–2023	0	0
	Lombardy	2021–2022	60	4
		2022–2023	9	81
	Piedmont	2021–2022	1	2
		2022–2023	0	2
South/Islands	Total	2021–2022	0	5
		2022–2023	0	2
Center	Total	2021–2022	5	1
		2022–2023	1	2
North-East	Total	2021–2022	251	11
		2022–2023	31	152
North-West	Total	2021–2022	61	6
		2022–2023	9	83

**Table 4:** Absolute frequencies (counts) of confirmed cases in wild birds, 2022–2023.

Species	Emilia-Romagna	Friuli-Venezia Giulia	Lombardy	AP Trento	Piedmont	Sardinia	Umbria	Veneto	Total
A	20	0	220	17	2	0	0	64	323
B	27	0	17	0	0	36	0	59	139
C	14	11	19	0	0	1	2	17	64
Total	61	11	256	17	2	37	2	140	526

**Table 5:** Location Index of confirmed cases in wild birds, 2022–2023.

Species	Emilia-Romagna	Friuli-Venezia Giulia	Lombardy	AP Trento	Piedmont	Sardinia	Umbria	Veneto
A	0.53	0.00	1.40	1.63	1.63	0.00	0.00	0.74
B	1.67	0.00	0.25	0.00	0.00	3.68	0.0	1.59
C	1.89	8.22	0.61	0.00	0.00	0.22	8.22	1.00

**Table 6:** Homogeneity Index by region.

Index	Emilia-Romagna	Friuli-Venezia Giulia	Lombardy	AP Trento	Piedmont	Sardinia	Umbria	Veneto	Total
HI	3.41	100.00	62.27	100.00	100.00	92.11	100.00	10.20	19.26

**Table 7:** Specialization Index by region.

Index	Emilia-Romagna	Friuli-Venezia Giulia	Lombardy	AP Trento	Piedmont	Sardinia	Umbria	Veneto
SI	28.62	87.83	24.53	38.59	38.59	70.87	87.83	15.72

## Data Availability

Data that support the findings of this study are available at https://zenodo.org/record/8246456 (accessed on 21 July 2023).
